# The Application of Nanoparticle-Based Drug Delivery Systems in Checkpoint Blockade Cancer Immunotherapy

**DOI:** 10.1155/2018/3673295

**Published:** 2018-09-30

**Authors:** Huan Zhao, Yuanqi Li, Dan Wei, Hongrong Luo

**Affiliations:** National Engineering Research Center for Biomaterial, Sichuan University, Sichuan, Chengdu 610064, China

## Abstract

Tumor is the most serious threat to human beings. Although war against cancer has been launched over forty years, cancer treatment is still far away from being satisfactory. Immunotherapy, especially checkpoint blockade immunotherapy, is a rising star that shows a promising future. To fulfill the requirement of depleting primary tumor and inhibiting tumor metastasis and recurrence, many researchers combined checkpoint blockade immunotherapy with other treatment strategies to extend the treatment outcome. Photodynamic therapy could induce immunogenic cell death, and checkpoint blockade could further accelerate the immunity; therefore, combining these two strategies publishes many papers. Additionally, photothermal therapy and immunotherapy were also utilized for combining with checkpoint blockade, which were also reviewed in this paper. Furthermore, antibodies, siRNA, and small molecule inhibitors are developed to block the checkpoint; therefore, we categorized the papers into three sections, combination nanoparticles with checkpoint blockade antibody, combination nanoparticles with checkpoint blockade siRNA, and combination nanoparticles with small molecule checkpoint inhibitors, and related researches were summarized. In conclusion, the combination nanoparticle with checkpoint blockade cancer immunity is a promising direction that may fulfill the requirement of cancer treatment.

## 1. Introduction

Tumor is the most serious threat to human beings. In China, the crude incidence rate of cancer was 278.07/100,000 [1]. Cancer is the leading cause of death in China and produces heavy burden to people [2, 3]. Until recently, major therapy strategies are still surgery resection, radiology, and chemotherapy. The outcome is limited because of the poor selection, high side effect, high ratio of metastasis, and recurrence. The development of nanotechnology provides powerful and functional nanoparticles that can deliver various drugs specifically into tumor, responsively release cargoes to tumor, and effectively exert antitumor effects to treat not only primary tumors but also metastasis and resident tumor cells after surgery [4]. The nanoparticles can be designed with various fancy properties, such as active tumor cell or stromal cell targeting, biological barrier-penetrating capacity, tumor microenvironment-responsive property alternation, and cargo release and external stimuli response or energy conversion capacity [4–9]. Although great achievement has been made, clinical translation and tumor heterogeneity are main obstacles for enlarging the tumor treatment outcome. A new therapeutic strategy is still urgently needed.

Normally, immunotherapy could recognize and destroy tumor cells by employing the patient's own immune system rather than exogenous toxicants. Immunotherapy is an attractive strategy because of their high specificity and efficiency [10]. However, the tumor microenvironment could produce immune-suppressive conditions that attenuate the immunity response. To enlarge the immunotherapy, three methods are developed, including cancer vaccines, adoptive cell therapy (e.g., CAR-T), and immune checkpoint blockade immunotherapy [11, 12]. Immune checkpoint blockade immunotherapy, first proposed in 2010, is a rising star that has gained great attention from both academy and industry [12].

Basically, tumor-specific T cells could kill tumor cells and inhibit tumor growth and metastasis. However, immune resistance or evasion shadows the outcome. Actually, immune resistance or evasion is a self-protection mechanism that could prohibit reorganization between T cells and normal cells by expression of specific checkpoints. However, the tumor cells may also express or secrete these checkpoints, leading to tumor immune resistance or immune evasion. Thus, immune checkpoints have been considered as novel targets for cancer immunotherapy [13, 14]. The programmed death 1 (PD-1) pathway and the cytotoxic T lymphocyte-associated protein 4 (CTLA4) pathway are two key targets in checkpoint blockade immunotherapy.

Antibodies are first developed for checkpoint blockade immunotherapy. Several PD-1, programmed death-ligand 1 (PD-L1), and CTLA-4 antibodies have been approved by the FDA for the treatment of advanced tumors, such asipilimumab, nivolumab, pembrolizumab, atezolizumab, and ipilimumab [15–17]. However, the antibodies are suffered by several disadvantages, such as high cost, low stability, and potential immunogenicity. Therefore, developing low-molecular-weight checkpoint inhibitors has been a new field in immunotherapy, and several inhibitors are reported [18]. Additionally, using siRNA direct knockdown PD-1 expression on tumor cells also could enlarge immunotherapy outcomes.

The key requirement for checkpoint blockade immunotherapy is the body already in high level of antitumor T cells, but the function was attenuated by specific checkpoints [13]. Therefore, many studies have used traditional therapy strategies to kill most of tumor cells, exert tumor immunity, and then combine with checkpoint blockade therapy to totally deplete the resident tumor cells and metastasis. Effective immunotherapy by checkpoint blockade or adoptive cell therapy is limited in most patients by the immunosuppressive tumor microenvironment. There is a variety of stromal myeloid and lymphoid cells in the tumor microenvironment, suppressing the activity of tumor-specific T cells. In this review, we will focus on the applications of nanoparticles in checkpoint blockade immunotherapy and categorize the studies by the checkpoint blockade antibody, siRNA, and small molecule inhibitor.

## 2. Combination Nanoparticles with Checkpoint Blockade Antibody

Nanoparticle-based chemotherapy, photothermal therapy, and photodynamic therapy showed promising antitumor effects by constructing fancy and intelligent systems that could actively target tumor cells and even specific organelles, such as nuclei and mitochondria, and responsively release cargoes to directly induce apoptosis of cancer cells or modulate the tumor microenvironment [7, 19, 20]. However, the inhibited immunity made the suppression of metastasis and recurrence not good enough. Therefore, direct combination nanotherapeutics with checkpoint blockade immunotherapy may further improve the antitumor effect.

### 2.1. Combination PDT with Checkpoint Blockade Antibody

**Figure 1:**
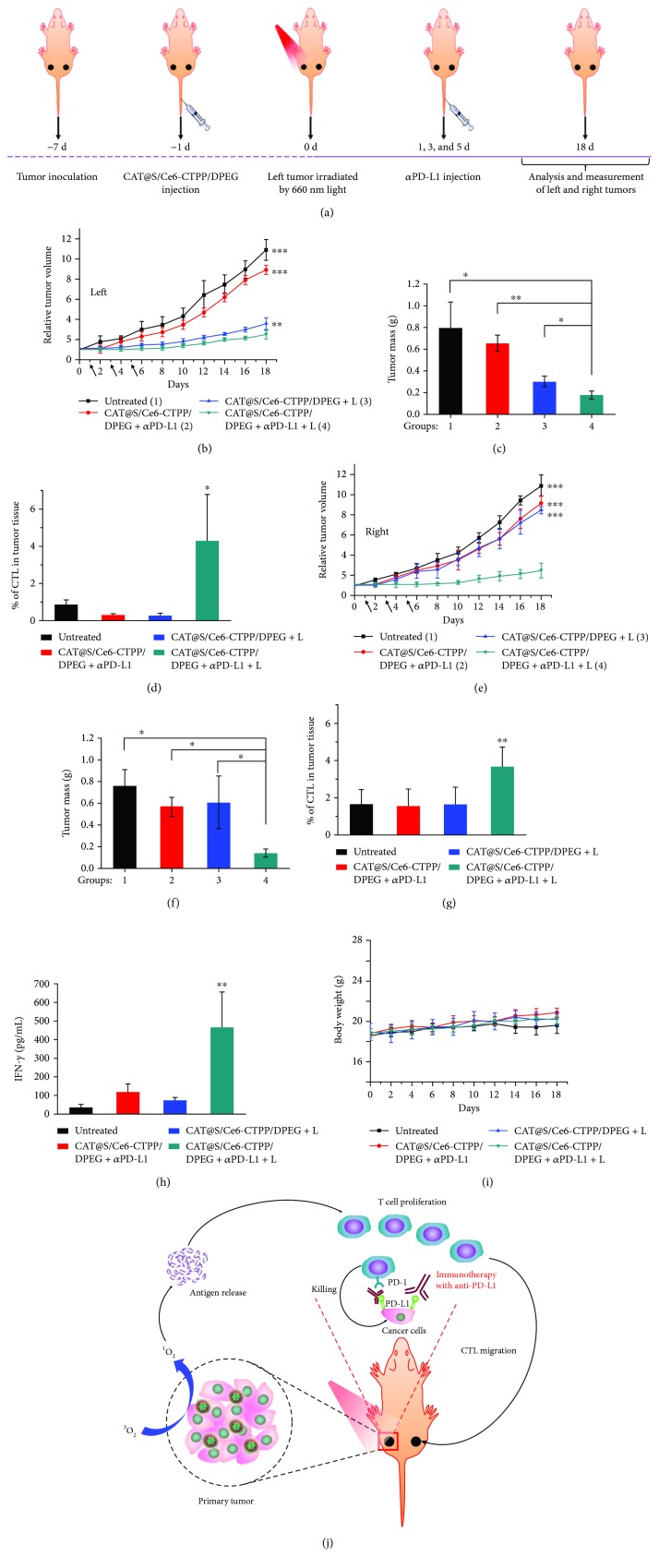
Abscopal effect of PDT with CAT@S/Ce6-CTPP/DPEG in combination with checkpoint blockade immunotherapy. (a) Schematic illustration to show the experimental design of combining PDT with anti-PD-L1 therapy (b–d). The tumor growth curves (b), average tumor weights at day 18 (c), and percentages of CTL infiltration at day 18 (d), for primary tumors (left) after various treatments indicated (e–g). The tumor growth curves (e), average tumor weights at day 18 (f), and percentages of CTL infiltration at day 18 (g), for nonirradiated tumors (right) after various treatments indicated. Data are presented as means ± standard deviations (*n* = 5). (h) The IFN-*γ* levels in sera from mice detected at 7 days after various treatments. (i) Changes in body weight of mice during treatment. (j) A scheme indicating the mechanisms of combining PDT with anti-PD-L1 therapy. *p* values in (b–h) were calculated by Turkey's post-test (^∗∗∗^*p* < 0.001, ^∗∗^*p* < 0.01, or ^∗^*p* < 0.05). The figure is adapted from [22] with permission of the copyright holder, American Chemical Society.

Nanoparticle-based photodynamic therapy (PDT) was commonly combined with checkpoint blockade immunotherapy because PDT could induce immunogenic cell death (ICD) [21]. Yang et al. constructed a multistage responsive drug delivery system based on hollow silica nanoparticles [22]. The nanoparticles were loaded with a hydrogen peroxide-decomposing enzyme to catalyze hydrogen peroxide to oxygen, and a photosensitive agent chlorine e6 (Ce6). The nanoparticles were modified with (3-carboxypropyl)triphenylphosphonium bromide (CTPP) for mitochondria targeting and then covered with an acidic pH detachable polymer that could be released in the tumor microenvironment and expose CTPP. The nanoparticles showed high accumulation in tumor cells and good mitochondrial targeting capacity. After injection, the oxyhemoglobin saturation level in tumor was increased from about 3% to 17%, demonstrating the nanoparticles with catalase which could decrease hypoxia of tumor, which was helpful for photodynamic therapy. As a result, the nanoparticles with light irradiation considerably inhibited the tumor growth; however, the nanoparticles showed no effect on other side tumors without light irradiation, suggesting the limitation of photodynamic therapy (Figure 1). To further improve the antitumor effect, Yang et al. combined the nanoparticles with intravenous injection of the PD-L1 antibody, resulting in significant growth inhibition of distant tumors, suggesting that the combination could promote infiltration of cytotoxic T lymphocytes into the distant tumors.

**Figure 2:**
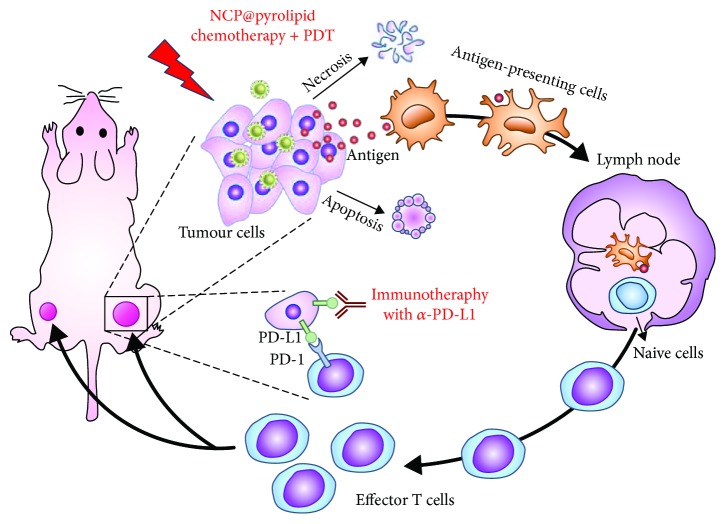
Chemotherapy and PDT of NCP@pyrolipid potentiate PD-L1 blockade to induce systemic antitumor immunity. Chemotherapy and PDT of NCP@pyrolipid induce ICD and an inflammatory environment at the primary tumor site, leading to the release of tumor-associated antigens (TAAs). TAAs are processed and presented by infiltrated antigen-presenting cells, to elicit the proliferation of tumor-specific effector T cells in lymphoid organs, such as tumor-draining lymph nodes. Combined with PD-L1 checkpoint blockade, NCP@pyrolipid chemotherapy/PDT significantly promoted the generation of tumor-specific effector T cells and enhanced their infiltration in both primary and distant tumors, resulting in not only tumor eradication in the primary sites but also a systemic antitumor immune response to reject distant tumors. The figure is adapted from [25] with permission of the copyright holder, Lin et al.

Duan et al. developed a core-shell nanoparticle (ZnP@pyro) with a photosensitizer pyropheophorbide a on the surface [23]. Calreticulin (CRT) is a specific marker expressed on the cell surface in response to ICD [24]. After in vitro PDT with ZnP@pyro, the expression of CRT was observed on about 78% of cells, and similar results were observed for in vivo PDT, suggesting that PDT could indeed induce ICD. Then, the PDT of ZnP@pyro was combined with PD-L1 antibody for the treatment of 4T1 tumor. Anti-PD-L1 treatment failed to delay tumor growth, and PDT led to a 75% reduction in tumor volume, while combination treatment completely eradicated the tumor, suggesting the good performance of PDT and anti-PD-L1 combinational therapy. Furthermore, combinational therapy could effectively inhibit the metastasis and inhibit the growth of preexisting metastasis. In a recent study, the photosensitizer was further combined with oxaliplatin for PDT and chemotherapy (Figure 2) [25]. The dual-loaded nanoparticles (NCP@pyrolipid) could effectively induce ICD of CT26 cells regardless of light irradiation because oxaliplatin itself could induce ICD. As a result, CRT is significantly expressed on the treated cells. When the treated cells inoculated into BALB/c mice, it could act as tumor vaccine to protect mice against challenge by live CT26 tumor cells. In vivo, NCP@pyrolipid showed high accumulation in tumor; it achieved 6.8% ID/g at 24 h post-injection, while the concentration in the liver was less than 7.1% ID/g. The treatment led to a significantly higher level of cytokines, such as TNF-*α* and IFN-*β*; thus, the growth of primary tumor was significantly inhibited. After combination with the PD-L1 antibody, the primary tumor was almost completely depleted, and the growth of distant tumor was also considerably inhibited, suggesting that the combination with checkpoint blockade immunotherapy could improve the antitumor effect both at primary and distant tumors. R837 and photosensitizer chlorin e6 (Ce6) dual-loaded upconverting nanoparticles were also developed for combination with the anti-CTLA-4 antibody [26]. Combination therapy could not only deplete primary tumor and tumor metastasis but also protect mice for a long time. It was shown that the effector memory of CD8^+^ T cells in spleen of the combinational therapy group was much higher than in other control groups, while the central memory of CD8^+^ T cells was much lower. After rechallenge, concentrations of both TNF-*α* and IFN-*γ* in mouse sera were significantly increased, suggesting that combinational therapy could boost a strong immune memory effect to prevent the recurrence of tumor.

To overcome the hypoxia condition of tumor and elevated PDT effect, Lan et al. developed a metal-organic framework (MOF) using Fe_3_O clusters and 5,10,15,20-tetra(p-benzoate)porphyrin (TBP) ligand (Fe-TBP) [27]. The Fe_3_O clusters can catalyze H_2_O_2_ in tumor hypoxia to O_2_, and then O_2_ could be excited to singlet oxygen by photo-excited porphyrins. In normal conditions, Fe-TBP showed an IC_50_ of 25.13 *μ*M for PDT, while IC50 of PDT with H4TBP was 11.33 μM. However, under hypoxic conditions, Fe-TBP still showed effective PDT with an IC_50_ of 3.10 μM. But H4TBP showed an IC_50_ higher than 50 μM. These results suggested that Fe-TBP could overcome the influence of the hypoxic condition on PDT. When combined with anti-PD-L1 antibody, Fe-TBP showed excellent antitumor effects that could completely deplete the subcutaneous tumor.

### 2.2. Combination Photothermal Therapy with Checkpoint Blockade Antibody

**Figure 3:**
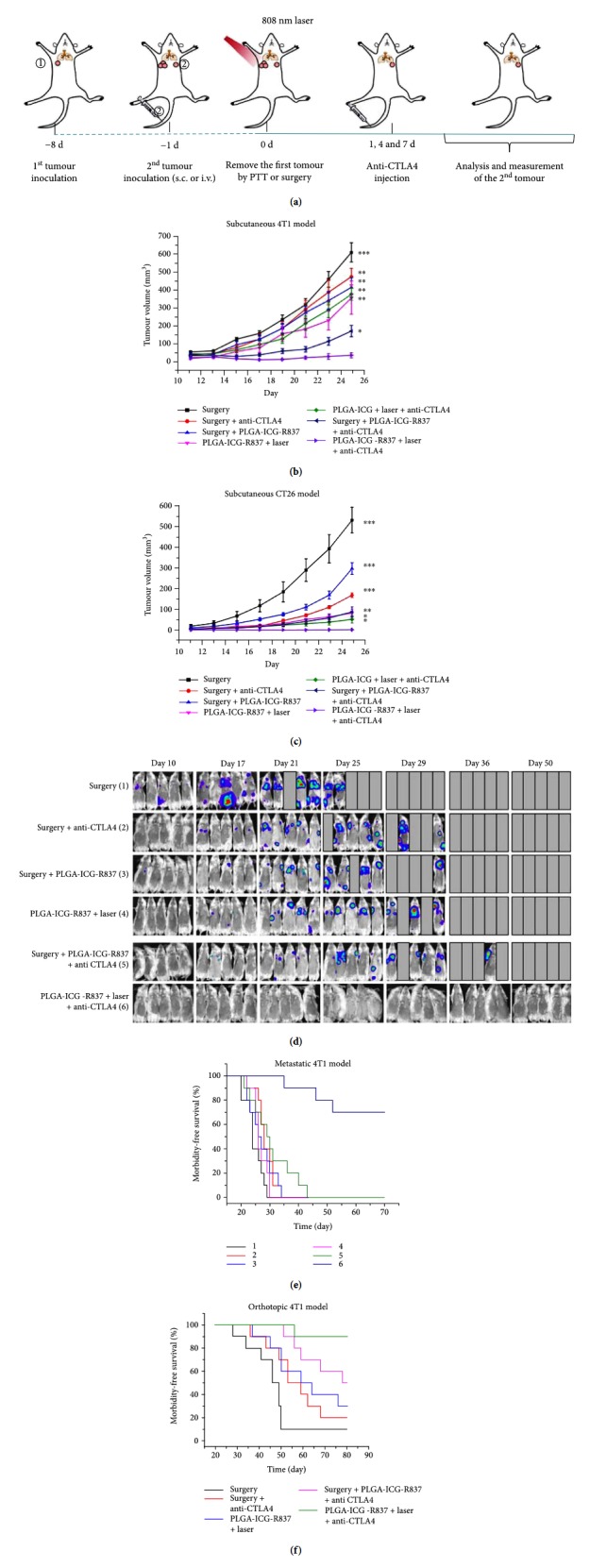
Anti-tumor effect of PLGA-ICG-R837-based PTT plus anti-CTLA-4 therapy. (a) Schematic illustration of PLGA-ICG-R837-based PTT and anti-CTLA-4 combination therapy to inhibit tumor growth at distant sites. (b, c) Tumor growth curves of different groups of mice (six mice per group) with s.c. inoculation of secondary 4T1 (b) or CT26 (c) tumors after various treatments to eliminate their primary tumors. (d) In vivo bioluminescence images to track the spread and growth of i.v. injected fLuc-4T1 cancer cells in different groups of mice after the cancer cells after various treatments to eliminate their primary tumors. (e) Morbidity-free survival of different groups of mice with metastatic 4T1 tumors in (d) after various treatments indicated to eliminate their primary tumors (10 mice per group). (f) Morbidity-free survival of different groups of mice bearing orthotopic 4T1 tumors with spontaneous metastases after various treatments indicated to eliminate their primary breast tumors (10 mice per group). PLGA-ICG-R837-based photothermal ablation of the first primary tumors in combination with anti-CTLA4 treatment would be able to induce strong anti-tumor immunological effects to inhibit the growth of tumor cells spreading into other organs. *p* values in (b) and (c) were calculated by Turkey's post hoc test (^∗∗∗^*p* = 0.001, ^∗∗^*p* = 0.01, or ^∗^*p* = 0.05) by comparing other groups with the last group (PLGA-ICG-R837þlaserþanti-CLTA-4). Data are presented as the mean ± s.e.m. The figure is adapted from [28] with permission of the copyright holder, Liu et al.

Photothermal therapy could directly induce apoptosis of tumor cells, and it can be combined with immune adjustment to elevate the immune response level. Chen et al. developed a poly(lactic-co-glycolic acid) (PLGA) nanoparticle to co-load indocyanine green (ICG) and imiquimod (R837) for photothermal therapy and activating immune responses because R837 was a Toll-like receptor 7 agonist [28]. In vivo, irradiation by an 808 nm laser for 10 min (0.5 W/cm^2^) could increase the temperature of tumor to about 60°C, which could almost completely ablate the primary tumor. After PTT, about 72% DC maturation was observed in tumor-draining lymph nodes, which was much higher than the adjuvant nanoparticles without laser irradiation, suggesting that DCs could be recruited to the tumor after PTT and act as antigen-presenting cells to trigger enhanced immune response. Then, PLGA-ICG-R837 was combined with cytotoxic T lymphocyte-associate antigen-4 (CTLA-4) antibody to evaluate the potential inhibition on metastasis. It was shown that the combination of PTT and CTLA-4 antibody could completely inhibit the growth of distant tumor and lung metastasis. In comparison, treatment with PLGA-ICG-R837 or CTLA-4 antibody could only slightly slow the growth of secondary tumor and the metastasis could still be observed apparently (Figure 3). The study indicated that combination checkpoint blockade immunotherapy with photothermal therapy could successfully inhibit the tumor metastasis, which provided a promising way for tumor treatment.

### 2.3. Combination Immunotherapy with Checkpoint Blockade Antibody

**Figure 4:**
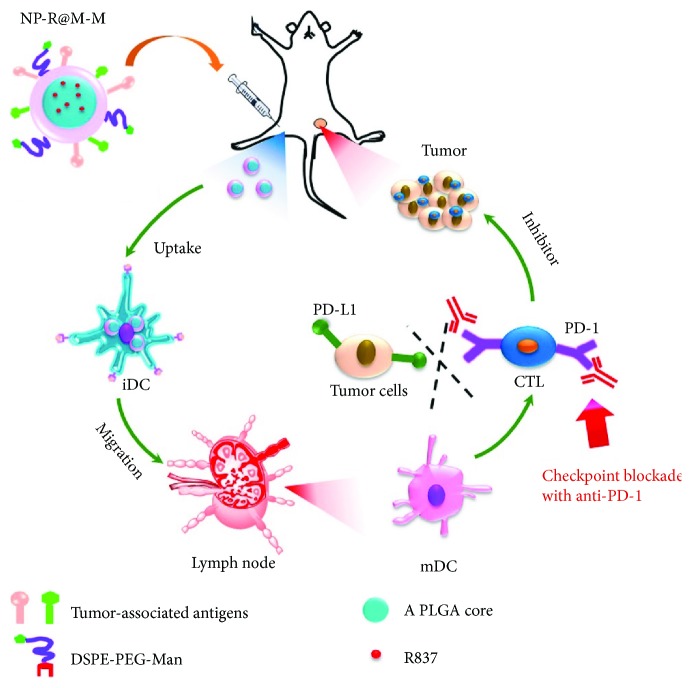
Schematic illustration to show the structure of tumor cell membrane-coated, R873-loaded, and mannose-modified PLGA NPs (NP-R@M-M) and their functions to induce antitumor immunity as a nanovaccine. The figure is adapted from [29] with permission of the copyright holder, American Chemical Society.

R837-loaded PLGA nanoparticles (NP-R) could serve as adjuvant for antigens presenting to DCs. To further improve the effect, Yang et al. coated the NP-R with cancer cell membranes and further modified with mannose moiety (NP-R@M-M) [29]. The particles with mannose showed a much higher uptake by bone marrow-derived DCs (BMDCs) than that without mannose, suggesting that mannose could elevate the targeting capacity to DCs. Furthermore, due to the overexpressed mannose receptors on macrophages, NP@M-M showed higher cellular uptake by macrophages than that of particles without mannose. The enhanced APC uptake of NP@M-M would be favorable for immune response induction. After being taken up, NP-R@M-M successfully stimulates the maturation of DCs. Moreover, the secretion of cytokines by BMDCs, such as tumor necrosis factor-*α* (TNF-α) and interleukin-12 (IL-12p40), was the highest level compared with other groups. In vivo, although intradermal immunization with NP-R@M-M significantly delayed the growth of B16-OVA tumor, it showed rapid growth later on. When combining the NP-R@M-M immunity and anti-CTLA-4 antibody, tumor proliferation could be effectively inhibited, and about half of the mice could survive at least 45 days free of tumor (Figure 4). The study demonstrated that combination of the nanovaccines with checkpoint blockade immunotherapy would be a promising method in treatment of tumors.

Regulatory T (Treg) cells can downregulate the activation and proliferation of immune cells to keep immune homeostasis [30, 31]. However, the enrichment of Treg cells in the tumor microenvironment responds to tumor progression, immune escape, and poor patient survival. The secretion of angiostatic cytokines was reduced, but the secretion of angiogenetic factors was enhanced [32]. Therefore, reduction of Treg cells in the tumor microenvironment may improve the tumor treatment. Imatinib could block STAT2 and STAT5 signaling that can decrease Treg cell abundance and attenuate their suppressive functions [33]. Ou et al. developed a kind of hybrid nanoparticle to load imatinib, and the tLyp-1 peptide was modified onto the surface to elevate the targeting capacity to Treg cells because its receptor neuropilin-1 was highly expressed on Treg cells but few expressed on T effector cells [34, 35]. In vivo, tLyp-1 modification significantly increased cellular uptake by neuropilin-1-overexpressed DU145 cells. Notably, in a coculture system, tLyp-1-modified nanoparticles showed much higher uptake in Treg cells than in CD8^+^ T cells, while no significant difference was observed in unmodified nanoparticles. In vivo, tLyp-1-modified nanoparticles showed higher accumulation in tumor after intravenous injection. After preparing the single-cell suspension from tumor, tLyp-1-modified nanoparticles showed higher capture in Treg cells than unmodified nanoparticles did, demonstrating the good targeting capacity of tLyp-1-modified nanoparticles to Treg cells. Treatment with imatinib-loaded tLyp-1-modified nanoparticles with anti-CTLA-4 antibody showed the best antitumor effect, while the combination index was 0.52, suggesting the synergistic effect of the combinational therapy. In the combinational therapy, the Treg cells in the tumor were decreased from 13.2% to 6.8%, the CD8^+^ T effector cells significantly elevated, and the secretion of proinflammatory cytokines IFN-*γ* and TNF-*α* was significantly increased [34]. These results demonstrated that combinational therapy could reverse the immunity suppression microenvironment of tumor and increase the immunotherapy outcomes.

The tumor cells undergoing ICD could release immunostimulatory signals to reverse immune tolerance and stimulate antitumor immune responses [36]. Fan et al. directly utilized the immunogenically dying tumor cells as antigens and then modified them with a CpG oligonucleotide-loaded nanodepot platform [37]. The CpG is a Toll-like receptor-9 (TLR9) agonist that could promote antigen presentation by antigen-presenting cells and prime CD8^+^ T cell response [38]. The whole-cell vaccines could be effectively presented to DCs and prime the CD8^+^ T cells to kill tumor cells. The vaccine could also release high levels of TNF-*α* and IFN-*β* to mediate inflammatory and immune response. In vivo, after CT26 tumor was established, a single dose of the whole-cell vaccine could significantly inhibit the tumor growth compared to PBS groups, while CpG-loaded nanoparticles showed no difference compared with PBS. To further amplify the antitumor effect, Fan et al. combined the whole-cell vaccine with anti-PD-1 IgG therapy. Combinational therapy significantly inhibited the tumor growth, and about 78% of the tumors were completed depleted. Importantly, combinational therapy showed a long-period immunity protection. At day 70, the mice could reject the rechallengement by CT26 tumor cells.

Wang et al. developed a kind of DNA nano-cocoons (DNC) that were assembled by long-chain single-stranded DNA and anti-PD-1 antibody, which can be loaded during the nanoparticle formation [39]. The DNA consists of repeated CpG sequences with an interval sequence that can be cut by restriction enzyme HhaI. The HhaI enzyme-loaded triglycerol monostearate (TGMS) nanoparticle was anchored onto the surface of DNC (TGMS-DNC) and can be released in the presence of esterases and MMPs which was overexpressed in tumor stroma and wound sites. In vitro incubation of TGMS-DNC with lipase or MMP-9 led to an effective release of CpG and anti-PD-1 antibody as determined by agarose gel electrophoresis and ELISA assay. Furthermore, the degradation of TGMS-DNC could effectively induce IL-6 and TNF-*α* production in RAW264.7 cells, suggesting that the functional CpG sequences were released. In vivo, after surgery resection of the subcutaneous B16F10 tumor, the particles were injected to the operative site, which overexpressed MMP-9 as determined by ELISA. The injection with dual-loaded TGMS-DNC resulted in the smallest relapsed tumor volume, while free anti-PD-1 antibody and free CpG nucleotide combination therapy only showed modest effect on delaying relapsed tumor growth. This study suggested that combination therapy could effectively reduce the relapse potential of tumors, while a sustained and responsive release of CpG and anti-PD-1 antibody is important for extending the outcome of combinational therapy.

Microneedles are developed for transcutaneous delivery of the anti-PD-1 antibody. When co-loading glucose oxidase and the anti-PD-1 antibody into pH-sensitive nanoparticles and fabricating them onto microneedles, glucose oxidase could convert glucose to gluconic acid, and then the acidic condition could trigger the release of the anti-PD-1 antibody for immunotherapy [40]. To further extend the immunotherapy, Ye et al. developed a microneedle delivery system to co-load the indoleamine 2,3-dioxygenase (IDO) inhibitor and anti-PD-1 antibody [41]. 1-Methyl-DL-tryptophan (1-MT), an IDO inhibitor, was conjugated to hyaluronic acid, and then it could form nanoparticles (HA-NPs) and encapsulate the anti-D-1 antibody. Finally, the nanoparticles were integrated into the microneedle system for transcutaneous immunotherapy of melanoma. The microneedle system could directly deliver the two drugs to the skin-resident dendritic cells around the tumor. Meanwhile, the system could achieve long-term release of the two drugs and the release could be triggered by hyaluronidase, which was overexpressed in tumor [20]. The diameter of HA-NPs could be decreased from 151 nm to 8 nm after incubation with 1 mg/mL of hyaluronidase for 24 h, demonstrating that the particles were hyaluronidase-sensitive. At the same time, the release of the anti-PD-1 antibody was significantly quicker in the presence of hyaluronidase than without hyaluronidase. Quantitative results suggested that the concentration of 1-MT in melanoma 1 day after treatment with the microneedle system was 3-fold higher compared with free 1-MT treatment, and more anti-PD-L1 antibody was captured in tumor cells by immunofluorescence staining. Furthermore, the blood circulation time of 1-MT was also significantly prolonged. For the in vivo antitumor study, the microneedle system contained only the anti-PD-1 antibody or 1-MT showed limited antitumor efficiency, while a considerable antitumor effect was observed in combinational therapy. 70% mice survived at day 40 after treatment.

## 3. Combination Nanoparticles with Checkpoint siRNA

**Figure 5:**
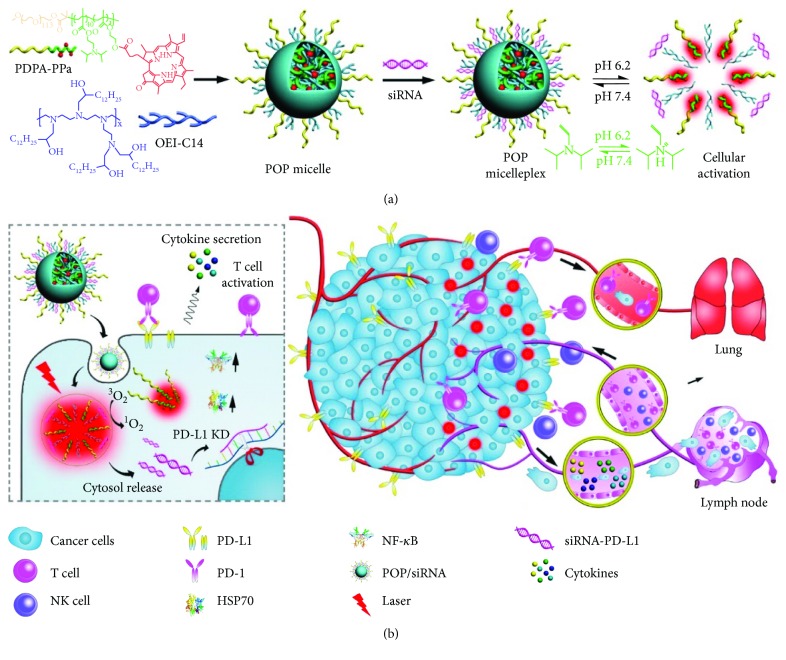
Schematic illustration of the acid-activatable micelleplexes for PD-L1 blockade-enhanced photodynamic cancer immunotherapy. (a) Chemical structure of the acid-activatable POP micelleplexes co-loaded with PPa and siRNA; the micelleplexes are dissociated at an acidic microenvironment due to the protonation of the tertiary amino groups of PDPA. (b) Cartoon schematic of the POP-PD-L1 micelleplex-mediated photodynamic cancer immunotherapy. The POP-PD-L1 micelleplexes induce ROS generation upon PDT. ROS consequently induces adaptive immune response by eliciting HSP70 and NF-*κ*B expressions, triggering proinflammatory cytokine secretion, and recruiting tumor infiltrating T cells. RNAi of PD-L1 can further enhance pDT-induced antitumor immune response. The figure is adapted from [43] with permission of the copyright holder, American Chemical Society.

The binding of PD-L1 on tumor cells with PD-1 on T cells could induce immune evasion due to the suppression of cytokine secretion, resulting in a compromised antitumor effect and metastasis [42]. Therefore, it is a promising method that directly knocks down the expression of PD-L1 on tumor cells. Wang et al. developed 1,2-epoxytetradecane alkylated oligoethylenimine-containing (POP) hybrid micelles to load PD-L1 siRNA and the photosensitizer [43]. The POP micelles could be taken up into tumor cells, escape from endosomes, and then release the siRNA for silencing PD-L1 expression on tumor cells (Figure 5). In vitro, 160 nM of siRNA-PD-L1 in POP micelles could considerably reduce over 50% expression of PD-L1 on B16F10 cells. In vivo, compared to PBS and POP controls, the POP + laser and POP-PD-L1 + laser groups elicited about 2-fold higher IFN-*γ* secretion. The frequency of tumor-infiltrating CD8^+^ T cells in the POP + laser and POP-PD-L1 + laser groups was 8.6- and 13.4-fold higher than in the PBS group, respectively. Furthermore, the frequency of CD4^+^ cells in the POP-PD-L1 and POP-PD-L1 + laser groups was 3.8- and 4.2-fold higher than in the PBS group, respectively, while the combination of PDT with PD-L1 knockdown could prompt the secretion of several proinflammatory cytokines and lead to a higher percentage of TNF-*α* and IFN-γ dual-positive CD8^+^ T cells. The cooperative activation of CD8^+^ T cells by PDT and PD-L1 knockdown could completely eliminate the B16F10 tumor, which was much better than single treatment. What is more, lung metastasis, a common compliant of B16F10 tumor, was completely inhibited.

Dai et al. developed a pH-sensitive charge-reversible PEI-conjugated poly(2-(diethylamino)ethyl methacrylate) (PDEA) copolymer (PEI-PDEA) and PEG block-conjugated PDEA (PEG-CDM-PDEA) [44]. In pH 7.4, the positively charged PEI-PDEA showed high binding affinity with PD-L1 siRNA and could form nanocomplexes with PEG-CDM-PDEA and a mitochondria-targeting photosensitizer MTPP. After entering into tumors by EPR effect, the acidic condition leads to the hydrolysis of the 2-propionic-3-methylmaleic anhydride (CDM) linker between PEG and PDEA; thus, PEG was detached and the surface charge changed to positive, facilitating cellular internalization. In the much lower pH condition of endosomes (about 5.0~5.5), PDEA would be protonated and the nanocomplexes were disassembled to release the PD-L1 siRNA and MTPP. MTPP could target mitochondria and induce phototoxicity to mitochondria under laser irradiation, while PD-L1 siRNA could knock down the expression of PD-L1 to improve the immunotherapy. As a result, the combination of PDT and gene therapy significantly elevated the antitumor effect and inhibited the metastasis of melanoma in mice. What is more, the survival ratio of mice after 30 days was 83%, which was much higher than in other groups.

Luo et al. developed folic acid- (FA-) modified polyethylene glycol-polyethylenimine (PEG-PEI) complexes with superparamagnetic iron oxide nanoparticle for PD-L1 siRNA delivery, and the SPIONs enabled the particles to be imaged by magnetic resonance imaging (MRI) [45]. FA modification significantly enhanced the uptake of the complex by folate receptor-overexpressed gastric cancer cells than the non-FA-modified control, and the gene transfection efficiency showed a similar trend. The T cell and cancer cell coculture system was used to evaluate the influence of particles on T cell function. It was shown that treatment with the FA-modified complex led to more cytokine secretion. Although the results were promising, no further in vivo experiments were provided.

## 4. Combination Nanoparticles with Small Molecule Checkpoint Inhibitor

Indoleamine 2,3-dioxygenase (IDO) is an immunoregulatory enzyme that can catalyze oxidation of tryptophan to hynurenine, which could inhibit the proliferation of T cells and induce apoptosis of T cells [46, 47]. Therefore, inhibition of IDO with inhibitors can enhance T cell-dependent antitumor immunity, and IDO inhibitors have gained increasing attention in new drug development [48]. Several small molecule inhibitors of IDO were developed and evaluated as new drug candidates. However, the phase II clinical trial failed recently. Lu et al. loaded an IDO inhibitor (INCB24360 analogue, 4-amino-N-(3-chloro-4-fluorophenyl)-N′-hydroxy-1, 2,5-oxadiazole-3-carboximidamide) to a chlorin-based nanoscale metal-organic framework (nMOF), TBC-HF [49]. The IDO inhibitor-loading capacity of constructed IDOi@TBC-HF was 4.7% and was slowly released from TBC-HF. The IC_50_ of TBC-HF with light was 5.48 *μ*M, which was much lower than that of protoporphyrin IX, an FDA-approved photosensitizer. After PDT, the MC38 cells showed significant calreticulin expression on the surface, indicating that PDT by TBC-HF could lead to immunogenic cell death (ICD). Since the released IDO inhibitor could modulate the suppressive tumor microenvironment of both the treated and untreated tumors, the abscopal effect was evaluated in colorectal cancer mouse models. After treatment, the volume of CT26 tumor was only 1.1% as that of the PBS group, while the size of distant tumor was also considerably decreased, indicating that PDT accompanied with the IDO inhibitor successfully excited systemic antitumor immunity.

Antibodies have several disadvantages, including high cost, poor stability, and potential immunogenicity [50]. Small peptides that can overcome the above-mentioned shortages show promising application in checkpoint immunotherapy. However, the peptides are suffered by poor proteolytic stability in serum and short blood circulation time [51]; therefore, sustained release of the peptide using nanoparticles gains great attention. Ma et al. used thermosensitive polymer poly(N-isopropylacrylamide) (PNIPAAm, P1) and peptide-conjugated poly(NIPAAmco-HEAm) (P3) as a temperature-sensitive nanophase-segregated surface to coat gold nanorods [52]. Compared with the insensitive control, the P1- and P3-coated nanorod (NR-2) showed negative increase in particle size in the presence of serum, but the size of P3-coated nanorod (NR-1) increased from 90 nm to 180 nm, suggesting that thermosensitive P1 polymer coating could inhibit protein binding, which is useful for extending blood circulation time [53]. As expected, the plasma half-life of NR-2 was about 2-fold longer than that of NR-1. Furthermore, free peptide could be completely digested within 8 h, and the peptide on NR-1 was digested by 59% within 48 h, while the peptide on NR-2 was only digested by 18% within the same time period, demonstrating that P1 and P3 polymer coating could protect the peptide drug. Both in vitro and in vivo, peptide-loaded NR-2 could effectively bind with B16F10 tumor cells and block the binding of the PD-L1 antibody. As a result, tumor growth was significantly delayed by treatment with peptide-loaded NR-2.

Luo et al. developed a PLGA nanoparticle to dually load the anti-PD-1 peptide and hollow gold nanoshell (HAuNS), while the latter one served as a photothermal agent [54]. After intratumor injection and laser irradiation, the temperature of tumor could quickly increase to 50°C in 3 min, indicating the good photothermal conversion ability. After photothermal therapy, the tumor growth was completely inhibited while the control group grew quickly. However, photothermal therapy showed minimum effect on the distant tumors. When combining with the anti-PD-1 peptide, the dual-loaded nanoparticles significantly inhibited the growth of distant tumors, suggesting that photothermal therapy with the anti-PD-1 peptide could induce strong immunity to kill the tumor cells distantly. Furthermore, the survival rate of tumor-bearing mice increased to 80% at day 30 after the first treatment.

The systemic blockade of immune coinhibitory signaling pathways could result in severe side toxicities. Song et al. recently reported the combination of immunogenic chemotherapy and locally expressed PD-L1 trap fusion protein for efficacious and safe cancer immunotherapy. They developed a unique trimeric PD-L1 trap protein by genetically fusing the extracellular domain of PD-1 with a robust trimerization domain from the cartilage matrix protein through an optimized hinge linker and demonstrated that oxaliplatin (OxP) boosts anti-PD-L1 mAb therapy against murine colorectal cancer. The local and transient expression of checkpoint inhibitors in the tumor microenvironment provides an ideal option to reduce these irAEs. Indeed, the combination of OxP and locally expressed PD-L1 trap did not induce the appearance of Th17 cells in the spleens as observed in the anti-PD-L1 mAb-treated mice, indicating that this strategy is a more efficient and safer option for cancer immunotherapy [55].

## 5. Conclusion and Perspective

Only 8 years passed since checkpoint blockade immunotherapy was proposed. During the development of novel antibodies, peptides, siRNA, and other inhibitors, many researchers have been trying to combine checkpoint blockade immunotherapy with other treatment strategies to further improve the antitumor effect. Until now, only limited studies were published. Although most of the studies showed promising results, there are still many unmet concerns, including the administration periods and intervals, off-target potentials, and host vs. host immunity responses. There is still a long way to go, but we believe there is a promising future of combinational therapy.
